# Permanency of the Teeth as Seen from a Study of Comparative Dental Anatomy

**Published:** 1904-07-15

**Authors:** Robt. B. Howell

**Affiliations:** Ann Arbor, Mich.


### PERMANENCY OF THE TEETH AS SEEN FROM A STUDY OF COMPARATIVE DENTAL ANATOMY.

BY ROBT. B. HOWELL, D.D.S., ANN ARBOR, MICH.

Read before the Detroit Dental Society, May 12, 1904.

Dentists are inclined to think that all that is possible is being done to effect the permanency of the teeth. The public is more than ever before being brought to a knowledge of the necessity of having their teeth cared for, and the dentist of to-day, by the possession of a superior education, is saving teeth which comparatively few years ago would have been extracted by his less fortunate brother. In fact so common has this *habit* of visiting dental offices become, however, every one may not agree with the writer in this statement, that we might well say that more teeth are being saved to-day than ever before in the history of dentistry. And again never was there so much done for the treatment of diseased gums and teeth, and for the correction of irregularities to maintain the full-sized normal jaw, than at the present time. The question arises, Does not the public rely too *much* upon the skill and ability of the dentist to relieve them of their troubles, rather than endeavor to do away with those erratic habits which cause dental diseases and deformities of various kinds? We might also inquire if the dentist is doing his full duty in an educational way.

As might be wrongly inferred from the subject, it is not the purpose of this paper to discuss whether or not we are descended from that simple and insignificant spindle-shaped vertebrate, the amphioxous, nor is it the intention to enter into a dissertation on Comparative Dental Anatomy, but simply to mention one or two truths which are discovered in the study of the latter and point out their practical significance to the profession.

In the study of all forms of vertebrates one cannot help noticing and admiring how perfectly they are adapted for the environment in which they exist. Take the fish, for example, how admirably it is suited for the aquatic life; the head is so shaped that it forms the least resistance to the water, there is no neck, the head being united directly to the body and held perfectly rigid, forming a most perfect cut-water. The integument is also peculiarly constructed for this aquatic existence, it constantly secretes a mucous which protects it from the action of the water and enables it to move with greater speed. The scales are arranged so as to offer the least resistance. The eye is flat on its anterior surface and focused for near vision. Then there is the truly wonderful respiratory system, the blood being oxidized as it passes through the gills which are constructed of highly vascular processes over which the water, containing oxygen, is passed. This most marvelous provision of nature, the appropriateness of all the organs of vertebrates to suit their surroundings, is seen in all other beings, such as the amphibians, reptiles, birds and mammals.

It is a well-known fact that any of these specialized organs may become rudimentary or entirely wanting if not used, this being brought about by a change of environment and consequently a change of habits. Darwin says, "Disuse at that period of life when an organ is chiefly used, which is generally at maturity, seems to have been the chief factor in causing organs to become rudimentary. It causes a lessened flow of blood to the part and thereby lessens normal and functional development. Continued disuse thus weakens and diminishes any part of the organism." There is another law which should be considered, that is when any part becomes useless, economy of growth enters in, and there will be no unnecessary expenditure of vital material in the construction of the useless part; the nutritive supply will go to some other organ where it is more needed.

A good example of this fact is shown in the whales, which are mammals that have taken to the water and become fish like in form and structure, the anterior limbs becoming like the pectoral fins of a fish and the hind limbs lost entirely. The most striking example in the life of one family is seen in that of the suidæ, or hogs. On the wild bear there are long and powerful limbs, which enable him to run with greater speed than a horse. The ears also are quite large and the sense of smell very acute, to enable him to scent and hear approaching danger, and since the naimal is required to *root* for a living, and also his chief means of defense is by his mouth, it becomes necessary for him to have developed a large and very muscular head and neck with a correspondingly powerful pair of jaws, containing the tusks and teeth characteristic of the boar. Now put this animal through a process of domestication and see what happens, he loses all the above characteristics, or at least, they pass through a state of degeneration; his legs become shorter and less muscular, his ears smaller, and undoubtedly his sense of smell less keen, and since through the kindness of man he is no longer required to root for a living, his tusks become much smaller and his jaws less muscular.

This specialization of parts is shown more strikingly in the organs of mastication probably than in any other, and as seen in the boar there will be marked degeneracy due to like causes.

If the mechanism for the reduction of food of the vertebrates be examined, it will be found that it is confined almost entirely to the oral cavity. In the lower forms the teeth are supported upon the various bones and cartilages in and about the mouth; they may be found in the thorax or even in the oesophagus, as seen in some of the snakes. In the higher forms they are supported on the maxilla and mandible.

This great diversity of form and characteristics of the teeth is due to two causes, first to food selection and secondly to jaw movements. There is no doubt that this variation is influenced by the qualities of the peculiar substances which are used by the animals for food. Each species requires a certain kind of service for the reduction of its food. As has been aptly said that "it is an adaptation of tools to material and not of material to tools." Proof conclusive of this fact is found by a study of the ancestors of various animals, comparing their teeth with those of their descendants which have been obliged to take up a different mode of life or not exist.

In fishes and snakes whose teeth are merely for retentive purposes, there being no mastication, they are the simple, conical form and usually extend backward in the mouth in order to more certainly secure their prey. In the herbivorous mammals the teeth are much enlarged laterally and strengthened so as to resist the lateral strain which arises from the grinding motion of the jaws of these animals. Lastly in the carnivora which have the simple up-and-down hinge motion, the teeth are developed vertically with long points or cusps, extending in the direction of the resistance to the greatest strain. The jaw movements also influence the teeth. In the herbivorous there is that peculiar grinding motion of these mammals and the tempero-mandibular articulation is peculiarly constructed for that purpose. In the carnivora there is the one simple vertical movement, and consequently the tempero-mandibular articulation is a hinge joint allowing the one motion. Now, in the examination and study of the order of primates, we find similar characteristics. The teeth are of such form as is peculiar to a mixed diet and are therefore termed omnivorous. The tempero-mandibular articulation is also of such a character as to allow both the vertical and lateral movements of the jaws. The food of aboriginal man was of a mixed character and his teeth were made for grinding, tearing and incising.

In view of these facts, what can be said of the human race? All the leading evolutionists of the day agree in the statement that the races of mankind are undergoing a rapid evolution. Man is making for himself a new environment. The old adage of "Early to bed and early to rise, makes a man wealthy, healthy and wise," is relegated to the past. In fact, it seems that a man to now, accomplish anything of importance or to achieve success must work a greater part of the night to do so. Less sleep and rest seem to be necessary. Where, as a business man of a few years ago, would go to his office and spend the entire morning reading over his mail and the rest of the day answering the same, he now arrives about nine o'clock, glances it over, dictates answers until about 10:30 or less and has it completed and in the return mail before the morning is over. This is necessary for him to exist in the business world. The man of twenty-five years ago would now be swamped the first day.

All this has its effect upon his physical development. Nature in her efforts to alter man's organization to keep pace with these rapid changes is doing her level best, and we cannot be surprised at some of the freak specimens of mankind which are occasionally produced. The increased amount of brain work or mental exercise has caused the development of the brain to an enormous extent, and this in turn requires a larger brain case, and through faulty habits of living, bolting and eating of pre-digested foods and lack of bodily exercise, the body does not get its proper amount of nutrition. In order to produce this enlarged brain case, it must be done at the expense of the lime salts which would go to the other portions of the osseous system and principally to the jaws. This may be proven by an examination and comparison of the jaws of apes with those of man, or even the latter with those of the lowest races of mankind, which will show a marked degeneration, both in size of jaws and number and size of teeth. In truth, the reduction of jaws seems to have kept pace in an inverse ratio with the development of the brain and consequent enlargement of the brain case. If the jaws of an infant ape and man be placed side by side, a striking resemblance will be noticed, but in the adults we find a prominent development in that of the ape, and a relatively unsatisfactory one in that of man.

Dr. Talbot and many others have made observations demonstrating that the human jaw is growing smaller all the time. This is also shown in the degeneration of the third molar.

To come to the practical part of this paper, "What is the dentist going to do, or can anything be done to better this condition of affairs?" A great deal can be done, and it is principally in the way of educating the public. Although the dentist has his practical procedures to carry out, he is also responsible both in a direct and indirect way. The necessary procedures might be classified under four general heads:

1st. Proper selection of food and mastication of same, both for infant and adult.

2nd. Proper care of the teeth. This embraces the mechanical cleansing by the aid of the brush and powder, and pick or thread, also chemical cleansing by means of antiseptic mouth-washes, and operative procedures of the dentist, which may be necessary for the cleansing of the teeth and eradication of caries.

3rd. Proper orthodontic procedures to maintain the full-sized and well-formed dental arch.

4th. Retention of the teeth, both deciduous and permanent, in the jaws for their full time.

A lengthy article could be written on any one of these four subjects, but this paper will simply present the thoughts under each head and they can be elaborated in the discussion if so desired.

It has been shown that man's food should consist of that which requires thorough mastication, and in order to accomplish this it will be necessary to eradicate two present great evils. The first is that of bolting the food and then dosing with some digestive remedy to assist the stomach in performing a function for which it is not organized. Dare it be predicted that if this continues, man of future generations will have developed a craw and gizzard like the birds, for the purpose of carrying out the function which has been taken from the teeth to their threatened degeneration.

The other equally serious habit is that of filling the stomach with a food which has been both masticated and digested. This can certainly be called the age of predigested foods. Men do not get up early enough, are too busy at noon, and too tired at evening to take the time to properly eat a meal.

The writer on one occasion heard something like the following remark from a man of more than average intelligence: "I wish the day would come when our food could be prepared in tablet form, so we would not have to waste so much time as is now done at meal hours." This seems to be a prevailing sentiment. What is to be done? This question can be answered by a very homely but suggestive phrase. Man must get back to the woods or at least to undigested food.

The time to commence this campaign of education is immediately after birth of the child. Give it the mother's milk if possible, which is its natural food; do not raise it on artificial foods, for the disturbances to the digestive apparatus of the infant, which are bound to occur, will inevitably exert an ill effect upon the structure of the still undeveloped crowns of the teeth. After the temporary teeth are erupted give the child a crust of bread occasionally to gnaw, if it gets it once, it will certainly cry for it again. When the age is reached that the child can be made to understand and reason, then teach in the home, school, and dental offices the importance of thorough mastication of the food, in order to give the teeth and jaws their full amount of exercise for growth, and to allow the saliva to do that portion of the digestion for which it is made. By so doing good habits will be formed which will more than likely be carried through life.

Under the second division, in order to have a perfectly-developed jaw, it is necessary to have a perfect dental apparatus, which cannot be, unless the gums are healthy and the teeth free from caries. For this requisite, it will be necessary then to take the best care of the teeth and mouth.

This will consist in a thorough cleansing of the teeth by the aid of the brush of the proper shape, correctly applied with a good powder, augmented by proper use of a thread or tooth-pick.

And since bacteria are the cause of such destructive diseases in the mouth, it is very essential that some good antiseptic mouth-wash be used; this should always be according to directions of the dentist.

Following this come the operative procedures for the cleansing of those deposits on the teeth which cannot be removed by the other methods and which have such a predisposing tendency to pyorrhea. Lastly is the importance of operative procedures on carious teeth.

The importance of the third division, "Orthodontic Procedures," is often overlooked. Although, as stated in the early part of this paper, that there is probably more of this kind of work being done than ever before, yet if the reason for doing the same could be ascertained, it would be found in the great majority of cases to be entirely for its esthetic value. Mothers do not like the looks of their children's teeth, consequently they get them straightened. The dentist of to-day has an important duty in teaching parents the necessity of having all mal-occlusion of the teeth corrected, in order to maintain the well-formed jaw and place the teeth in the best possible position to perform their functions in the most effective manner and escape caries. The fact that this adds to the external appearance of face should be one of the least considerations.

The last division, "The Retention of the Teeth," is one which has been much discussed and written upon. There is, however, hardly anything which can be said in support of the extraction of the teeth, the most common being the first molars. The prevailing argument to support it is the best which can be used against it. Are these words familiar? "If the first molar be extracted before the twelfth year, the space will entirely fill up by the second molar moving forward and the teeth in front giving back slightly." This is exactly what is not wanted; it is this enforced contraction of the jaws which produces a deplorable state of affairs. Leave all the teeth in as long as anything can be done to save them; fill with amalgam, or cement or crown, anything to maintain the full number of teeth.

It is hardly necessary to mention the exigency of retaining the temporary teeth, so as to insure a regular permanent set. Fill them even if it is necessary to remove the pulp and fill the pulp cavities. It is better, however, to watch them carefully and endeavor to fill the cavities while they are small, especially cavities on the proximal surfaces.

If the average dentist of to-day carries out the outline as given in the above four divisions to the best of his ability and judgment, there will be no cause for alarm for the dental apparatus of future generations, and much will be done to elevate the profession to as high a standard as could have been desired by him whose place the writer is so feebly occupying this evening.

